# Benzene as a Chemical Hazard in Processed Foods

**DOI:** 10.1155/2015/545640

**Published:** 2015-02-18

**Authors:** Vânia Paula Salviano dos Santos, Andréa Medeiros Salgado, Alexandre Guedes Torres, Karen Signori Pereira

**Affiliations:** ^1^Laboratório de Sensores Biológicos, Escola de Química, Universidade Federal do Rio de Janeiro, Avenida Horácio Macedo 2030, CT, Bloco E, Sala E-122, Ilha do Fundão, 21941-598 Rio de Janeiro, RJ, Brazil; ^2^Laboratório de Bioquímica Nutricional e de Alimentos, Instituto de Química, Universidade Federal do Rio de Janeiro, Avenida Athos da Silveira Ramos 149, CT, Bloco A, Sala 528A, Ilha Fundão, 21941-909 Rio de Janeiro, RJ, Brazil; ^3^Laboratório de Microbiologia de Alimentos, Escola de Química, Universidade Federal do Rio de Janeiro, Avenida Horácio Macedo 2030, CT, Bloco E, Sala E-104, Ilha do Fundão, 21941-598 Rio de Janeiro, RJ, Brazil

## Abstract

This paper presents a literature review on benzene in foods, including toxicological aspects, occurrence, formation mechanisms, and mitigation measures and analyzes data reporting benzene levels in foods. Benzene is recognized by the IARC (International Agency for Research on Cancer) as carcinogenic to humans, and its presence in foods has been attributed to various potential sources: packaging, storage environment, contaminated drinking water, cooking processes, irradiation processes, and degradation of food preservatives such as benzoates. Since there are no specific limits for benzene levels in beverages and food in general studies have adopted references for drinking water in a range from 1–10 ppb. The presence of benzene has been reported in various food/beverage substances with soft drinks often reported in the literature. Although the analyses reported low levels of benzene in most of the samples studied, some exceeded permissible limits. The available data on dietary exposure to benzene is minimal from the viewpoint of public health. Often benzene levels were low as to be considered negligible and not a consumer health risk, but there is still a need of more studies for a better understanding of their effects on human health through the ingestion of contaminated food.

## 1. Introduction

Benzene (C_6_H_6_, CAS number 71-43-2) is an aromatic hydrocarbon widely used as a solvent in chemical laboratories and as an intermediate in the chemical industry for manufacturing of polymers and other products [1]. It is a common contaminant in the atmosphere. Roughly 99% of the benzene present in the human body was inhaled. Benzene in air comes either from natural sources, such as forest fires and volcanic activity, or from human activities, such as cigarette smoking and burning of fossil fuels [2]. Benzene is classified by the International Agency for Research on Cancer (IARC) as a Group 1 human carcinogen [3]. Nonsmokers inhale roughly 200–450 *μ*g of benzene a day, while smokers may inhale three times as much [4]. Most of the human exposure to benzene occurs by inhalation but consumption of contaminated foods and water also play a role. Benzene can enter foods in many ways: use of contaminated raw materials and packaging materials, storage in contaminated areas, and use of contaminated water for washing or preparing the food. It may form in food by some cooking processes, thermal decomposition of food components, additives, food irradiation, and preservative decomposition such as benzoate decomposition [5, 6]. Agricultural products may also be contaminated with benzene from emissions caused by fires and the use of fossil fuel-burning equipment [7]. Finally, benzene may form in some food additives, such as liquid smoke, during their manufacturing. Liquid smoke is made from partial wood combustion [8].

In 1993, Gardner and Lawrence [9] found that benzene can form when benzoate is decarboxylated in the presence of ascorbic acid and transition metals such as Cu(II) and Fe(III) and can be accelerated by light and heat [10]. Benzoate and ascorbic acid are widely used as preservatives and antioxidants, respectively, in nonalcoholic beverages but they may also occur naturally in foods [11].

Transition metals such as the ones mentioned above may catalyze the transfer of one electron from ascorbic acid to oxygen, producing the anion superoxide, which then undergoes spontaneous dismutation producing hydrogen peroxide. The further reduction of hydrogen peroxide by ascorbic acid is also catalyzed by those metals. This reduction may generate hydroxyl radicals, which can decarboxylate benzoic acid and form benzene [9].

Most studies indicate nonalcoholic beverages as the best candidates for benzene formation since ascorbic acid and sodium, potassium, or calcium benzoate are usually present in their composition.

Nutritive sweeteners such as sugar and high-fructose corn syrup, ethylenediaminetetraacetic acid (EDTA), and sodium hexametaphosphate may reduce or even inhibit benzene formation, increasing the susceptibility of diet and light nonalcoholic beverages to benzene formation [12, 13]. The ability of EDTA and sodium hexametaphosphate to chelate Cu(II) and Fe(III) ions may be reduced by competition, that is, by adding calcium or other minerals to the food. Another important factor is storage time: the formation of benzene is greater when foods are stored for long periods of time under high temperatures [10, 11, 14].

In 2006, the American Beverage Association [12] published some guidelines to help beverage manufacturers to reduce or inhibit the formation of benzene in their products. The most important guidelines involve replacing ascorbic acid by another antioxidant, adding EDTA or sodium polyphosphates to chelate the metalic ions that catalyze hydroxyl radical formation, and reviewing the storage conditions and shelf life to minimize product exposure to high temperatures and ultraviolet (UV) light [10].

There are no legal limits for benzene in foods or beverages. The limit established for potable water is used as reference: 10 ppb established by the World Health Organization (WHO), 5 ppb by the United States (USA) Environmental Protection Agency, and 1 ppb by the European Council [11, 15].

In 2011, the Brazilian Federal Public Ministry (MPF) signed a conduct adjustment term (TAC) with three soft drink manufacturers (Coca-Cola Indústrias Ltda., Companhia de Bebidas das Américas (AMBEV), and Primo Schincariol Indústria de Cervejas e Refrigerantes Ltda.) establishing a maximum acceptable benzene content of 5 ppb [16].

In this review, we summarized the benzene formation mechanisms in food and beverages, occurrence, and mitigation measures as well as data analysis of benzene in food (mainly nonalcoholic beverages) to investigate the potential risk of benzene exposure through oral ingestion.

## 2. Occurrence of Benzene in Foods

### 2.1. Food Processing

Benzene may be introduced in foods during processing, in processes like smoking, roasting, and ionizing radiation [17]. Not much information is available on smoking, except for a study that found benzene concentrations of 21 and 121 parts per billion (ppb) in smoked meats [8, 18].

Benzene in foods may form when the amino acid phenylalanine is broken down by ionizing radiation [19, 20]. While studying the mechanism of benzene formation in carrot juice for children, Lachenmeier et al. found that some substances, such as *β*-carotene, phenylalanine, or terpenes, may decompose during processing to yield benzene [21].

Six samples of beer have been contaminated with benzene through the use of contaminated carbon dioxide during carbonation [22].

### 2.2. Reaction between Benzoic Acid or Benzoates and Ascorbic Acid

Because of the growing demand for processed foods, preservatives have been gaining importance in modern food technology. Benzoic acid and its sodium and potassium salts are among the most common preservatives used for inhibiting microbial growth because of their cost-benefit ratios [23]. Benzoates control the growth of some bacteria, filamentous fungi, and yeasts, preventing product deterioration and the growth of pathogenic species [24].

Although undissolved benzoic acid is the most effective antimicrobial agent, benzoates are used more often because of their greater solubility in water. Benzoic acid has low solubility in normal temperatures [25]. A solution of benzoic acid contains dissolved and undissolved molecules in equilibrium. As the pH decreases, the equilibrium shifts towards undissolved benzoic acid [26].

Sodium, potassium, and calcium benzoates and ascorbic acid may occur naturally in foods or be added as food additives. Ascorbic acid usually occurs naturally in some fruit juices, such as orange, lime, and acerola, among others, and may also be added as an antioxidant or nutrient. Some benzoates/benzoic acids are present in some fruits and may be added as preservatives, especially in acidic foods with a pH < 4.5, which is usually the case of nonalcoholic beverages [26].

Transition metals, such as Cu(II) and Fe(III), may catalyze the transfer of one electron from ascorbic acid to oxygen, producing the radical superoxide, which then undergoes spontaneous dismutation to form hydrogen peroxide. Hydrogen peroxide is further reduced, a reaction also catalyzed by those metal ions, and may generate hydroxyl radicals [27].

In 1965, Matthews and Sangster [28] found that irradiation of an aqueous solution of sodium benzoate causes hydroxyl radicals to attack benzoate ions forming unstable benzoic acid radicals which easily lose the carbon dioxide attached to the aromatic ring to form benzene.

At the beginning of the 1990s, Gardner and Lawrence [9] showed that hydroxyl radicals generated by the reduction of oxygen or hydrogen peroxyde by ascorbic acid, catalyzed by metal ions, could decarboxylate benzoic acid through a pH-dependent reaction, resulting in the benzene formation in foods and beverages. The said reaction was favored by an acidic pH but significantly slowed down at the 3–5 pH range. Study of the effects of different pHs and concentrations of ascorbic acid, hydrogen peroxide, and metal ions showed that hydroxyl production is directly proportional to the concentration of ascorbic acid. However, when the concentration of ascorbic acid exceeds that of benzoic acid, the formation of hydroxyls decreases, since benzoic and ascorbic acids compete. Benzene formation increased linearly with hydrogen peroxyde concentration, until the ascorbic acid concentration was exceeded. At a temperature of 25°C and reaction time of 15 minutes, benzene did not form in the absence of ascorbic acid or hydrogen peroxide. But at a temperature of 50°C and reaction time of 3 hours, 25.0 ± 1.4 nM of benzene formed in a solution with a pH of 3, buffered with 50 mM phosphate and containing 8 mM ascorbic acid, 6.25 mM benzoic acid, and 0.25 mM CuSO_4_. Metal ions are important for the formation of hydroxyl radicals. Optimal benzene formation occurred in the presence of 1.0 mM CuSO_4_ and 0.05 mM FeSO_4_. Higher metal ion concentrations reduced benzene formation.

Analysis of more than 50 foods, including eggs, by purge-and-trap/static headspace concentration and capillary gas chromatography showed that foods without added benzoates had less than 2 ng/g of benzene. On the other hand, the concentration of benzene in foods with benzoates and ascorbates varied from 1 to 38 ng/g [18]. Benzene may form in foods and beverages with benzoic and ascorbic acids because the conditions simulated by Gardner and Lawrence [9] are the most prevalent during processing and storage. Raw materials, such as some fruits and condiments, may contain ascorbic acid, sodium benzoate, or antioxidants naturally, which may lead to varying concentrations of benzene in the final product [10, 18, 29, 30].

## 3. Factors That Influence the Formation of Benzene in Foods

Liquid model systems and food samples containing sodium benzoate and ascorbic acid have been used by many studies to verify benzene formation and the influence of intrinsic factors, such as raw materials, pH, concentrations of sodium benzoate, ascorbic acid, and metal ions; chelating action of antioxidants and sugars; and hydroxyl precursors. Extrinsic factors such as temperature, UV radiation, and storage time were also investigated.

McNeal et al. [18] used aqueous models containing 0.025% ascorbic acid and 0.04% sodium benzoate, the same concentrations used in processed beverages, to study the effect of temperature and UV on benzene formation. A total of 300 ng of benzene per gram of solution formed in models stored at 45°C or under intense UV light (wavelength and light intensity not reported) for 20 hours. On the other hand, only 4 ng/g of benzene per gram of solution formed in models stored in the dark and at room temperature for 20 hours. However, after 8 days, the benzene concentration had risen to 266 ng/g of solution.

Chang and Ku [31] conducted a study similar to that of McNeal et al. [18] using the same ascorbic acid and sodium benzoate concentrations but did not add copper and iron ions to the solutions. After eight days stored in the dark, the solutions had a benzene concentration of 176 ng/g. These results suggest that traces of metal ions present in the water or reagents may be enough to catalyze hydroxyl formation [17]. The addition of chelators, such as 0.1 mM EDTA and diethylenetriaminepentaacetic acid (DTPA), prevented benzene formation; 100 mM ethanol solutions decrease benzene formation by 90% [31].

Aprea et al. [11] studied the influence of temperature on benzene formation using aqueous models with the same ascorbic acid and sodium benzoate concentrations as those found in processed beverages. The solutions were stored at 25°C or 45°C for 72 hours. Benzene formation in the solution stored at 25°C remained constant for the first 12 hours (<0.1 *μ*g/L) but increased to 0.44 *μ*g/L after 70 hours; in the solution stored at 45°C, 118.5 *μ*g/L of benzene formed after 24 hours, increasing to 125 *μ*g/L after 48 hours. In work by Morsi et al. [32], soft drink samples were incubated at 3 different temperatures (20°C, 45°C, and 90°C) for 21 days prior to the determination of benzene and that resulted in a significant increase in the levels of benzene in samples subjected to temperatures of 45°C (ranging from 5.5 ppb to 6.6 ppb) and 90°C (ranging from 25 ppb to 55.1 ppb) compared to the stored samples 25°C (ranging from 0.7 ppb to 1.5 ppb). These data corroborate McNeal et al. [18] who tested liquid models under similar conditions.

Nyman et al. [10] investigated the influence of accelerated thermal conditions (40°C or 60°C for 24 hours or 40°C for 15 days) and UV light (known wavelength and intensity for 24 hours or seven days) on liquid models containing 0.025% ascorbic acid and 0.04% sodium benzoate or processed beverage samples containing one or more benzene precursors (0.04% sodium benzoate and ascorbic acid are added to cranberry juice). Polyethylene terephthalate (PET) bottles stabilized with Tinuvin (UV light filter) were used to determine how well this UV light filter inhibits benzene formation. Benzene formation was greater in samples heated to 40°C than in those exposed to UV or visible light. However, stabilized PET bottles reduced benzene formation in beverages containing orange juice only marginally. Among the samples not exposed to UV light or heat, only those with cranberry juice contained benzene (2.6 ng/g) after the storage period, possibly because benzoic acid is naturally found in cranberries.

Casado et al. [30] studied pickled green olives, cucumbers, and caper berries containing ascorbic and benzoic acids under three experimental conditions to determine the chemical and sensory changes induced by these additives: (0) without additives; (1) with sodium benzoate; and (2) with sodium benzoate and ascorbic acid. The samples were stored in glass or plastic bottles and under room temperature (20°C–24°C) or refrigeration (6°C–9°C). Benzene only formed in treatment 2 under storage at room temperature and plastic pouches pack. For refrigerated or short storage periods (<14 weeks), benzene concentrations were similar to those found by McNeal et al. [18] for pickled vegetables. Green olives stored in plastic bottles had 1.5 *μ*g/L of benzene at the end of a 67-week period. Depending on the vegetable matrix and packing material, benzene concentration in samples stored for more than one year can exceed 10 *μ*g/L, which is the maximum acceptable concentration of benzene in water according to the WHO [4]. Pickled cucumbers stored in plastic bottles had 44.7 *μ*g/L of benzene after 67 weeks, while pickled cucumbers stored in glass bottles had 5.2 *μ*g/L after 27 weeks. Pickled caper berries had lower benzene concentrations at the end of the storage periods, clearly demonstrating the influence of raw materials on benzene formation, possibly because some of these raw materials contained phenolic antioxidants, corroborating Medeiros Vinci et al. [33]. Even after all the ascorbic acid had reacted, as seen in pickled caper berries stored in plastic bottles for 26 and 27 weeks, benzene continued to form, indicating that benzoate decarboxylation can be induced by ascorbic acid degradation products during long storage periods at room temperature [30]. This suggests that both ascorbic acide and its degradation products may induce peroxide formation in the presence of metal ions and oxygen, generating hydroxyl radicals that attack benzoate, yielding benzene [9].

Medeiros Vinci et al. [33] investigated some factors that promoted benzene formation in liquid models containing benzoate. The said factors include the buffer system used, other sources of hydroxyl radicals in foods (riboflavin photooxidation and lipid oxidation), concentration of transition metal ions, and the inhibitory action of antioxidants. Benzene formation was greatest (1250 ± 131 *μ*g/kg^−1^) in the model containing riboflavin, ascorbic acid, and Cu(II) ions in a solution buffered with sodium citrate and exposed to light. Benzene formation was mostly attributed to the combined presence of ascorbic acid and transition metal ions at a concentration of 50 *μ*M (1400 *μ*g/kg^−1^). The study antioxidants were capable of reducing the formation of benzene, and when compared with EDTA, the standard antioxidant, which completely inhibited benzene formation at a concentration of 1 mM, would need to be present in higher concentrations to achieve the same results.

EDTA prevents the formation of benzene by chelating metal ions. Complexation inactivates the catalytic activity of the metal by occupying all of its coordination sites [34]. Calcium and other minerals present in food, especially beverages, may compete with copper and/or iron ions for EDTA, reducing EDTA's ability to prevent benzene formation. This is because hydroxyl radicals, which attack benzoic acid to yield benzene, only form in the presence of free copper and/or iron ions [17].

Antioxidants may occur naturally in foods or be added intentionally to prevent oxidation and the formation of hydroxyl radicals. They act through many different mechanisms, such as by scavenging metal ions or reactive species, and by donating a hydrogen atom to stabilize these species. In high concentrations, antioxidants may become a prooxidant and promote benzoate decarboxilation. The presence of other antioxidants and pH may also play a role [33].

In 2008, Aprea et al. [11] studied the effect of sugar on benzene formation in liquid models containing ascorbic acid, benzoate, and three different concentrations of sucrose (0.1, 0.25, and 0.5 M). Increasing sucrose concentrations inhibited benzene formation accordingly. The influence of other sugars, such as fructose and glucose, on benzene formation was also studied. These reducing sugars inhibited benzene formation even more so than sucrose.

## 4. Analysis of Benzene in Foods

Studies of benzene in food, especially in nonalcoholic beverages, are done globally (Table 1).

## 5. Dietary Exposure to Benzene

It is difficult to assess accurately human dietary exposure to benzene because benzene concentration in foods varies greatly [35]. The Human Exposure Characterization of Chemical Substances (HEXPOC) [36] has estimated that human dietary exposure to benzene varies from 3 to 50 ng/kg of body weight per day.

According to Smith et al. [35], the MoE (margin of exposure—2 · 10^6^ to 0.4 · 10^4^) was established for benzene dietary exposure based on dose-response modeling for benzene gave a BMDL10 for female Zymbal gland carcinoma of 17.6 mg/kg-bw/d. These authors believe that the amounts of benzene found in foods and beverages are too low to cause problems but emphasized that more studies are needed.

Lachenmeier et al. [37] used the margin of exposure (MoE) to calculate the exposure of children to benzene in carrot juice and found that there was no reason to avoid the occasional consumption of this beverage; however, one must bear in mind that this is not the only source of benzene exposure and that benzene tends to accumulate in the human body.

Another study found high MoE values, indicating low risk to public health [15]. Inhalation is the most common route of exposure to benzene but only 50% of the inhaled benzene is absorbed, while 100% of the benzene in foods and beverages is absorbed. Therefore, foods susceptible to benzene formation, such as processed foods, must be studied and monitored [15].

## 6. General Toxicological Characteristics

The lethal oral dose of benzene for humans is 125 mg/kg. Chronic exposure to low concentrations of benzene is associated with genotoxicity and damage to the hematopoietic system. According to carcinogenicity studies in rats, the reference value of benzene inhalation is 0.01 mg/L for an upper-bound excess lifetime risk of leukemia of 10^−5^ [38]. Benzene is classified by the International Agency for Research on Cancer (IARC) as a Group 1 carcinogen and its role as a leukemogen has been clearly established by a series of epidemiological studies [39].

Studies indicate that individuals exposed to 1-2 ppm of benzene, or even less, for 40 years may have an increased risk of experiencing its toxic effects, including leukemia [40].

Children are at high risk of both acute and chronic exposure to environmental contaminants. They are more vulnerable because they absorb more contaminants than adults exposed to equal concentrations. Children are estimated to intake 2.3 times more air and 6.1 times more food than adults per kilogram of body weight [41, 42].

## 7. Suppression of Benzene Formation

Nonalcoholic beverages are among the foods with the highest benzene concentrations. The amount of benzene found in other foods by qualitative and quantitative studies is small. Hence, the industry concentrates its efforts to reduce the formation of benzene on nonalcoholic beverages containing ascorbic acid and sodium benzoate [17].

Some guidelines have been established by the American Beverage Association in 2006 [12] to minimize benzene formation in beverages and recommended special attention to some points of the manufacturing process:

- Raw water may be contaminated with benzene.
- Sugars may reduce benzene formation but do not inhibit it completely. Therefore, light/diet products are more vulnerable to benzene formation.
- Both ascorbic and benzoic acids may occur naturally in juices because they are present in many fruits.
- Raw carbon dioxide may be contaminated—the limit is 20 ppm of benzene (v/v).
- When acidity is low, ascorbic acid together with sources of benzoic acid is very likely to produce benzene.
- Coloring agents and flavors may contain ascorbates.
- Consider removing, reducing, or replacing benzoates with other microbial growth inhibitors.
- Consider removing, reducing, or replacing ascorbates with other antioxidants.
- Check the product storage conditions since strong light and high temperatures speed up the formation of free radicals.
- Transition metals may be present in raw water and sweeteners. Traces of copper and iron may catalyze reactions involving ascorbic and benzoic acids. In this case, the addition of chelators, such as EDTA or sodium polyphosphates, may help to minimize benzene formation.

Nyman et al.'s study [49] showed contents of beverages benzene above 5 ng/g were reformulated by the manufacturer. Reformulation included such methods as removal of ascorbic acid, addition of EDTA, deletion of benzoates, and addition of a blend of sodium benzoate with potassium sorbate. All of the reformulated products were found to contain 1.1 ng/g benzene or less.

## 8. Conclusion

From the data presented in the literature and presented here, we conclude that benzene is a chemical hazard in food, although oral exposure is negligible compared to environmental exposure. Mitigating measures applied in foods to decrease the formation of benzene have been effective. The reduction in the concentration of precursors (benzoic acid and ascorbic acid) has been shown to be the primary measure used in the formulation of food and efficiently reduces the levels of contaminants in the final product; replacement of the antimicrobial agent (benzoate) is always an alternative to be considered. However, further studies on the actual prevalence of benzene in food samples, the intrinsic and extrinsic factors that actually contribute to their formation in food, and development/adaptation of methodologies for their detection in such matrices are needed. Finally data from studies of oral exposure to benzene are scarce and such experimental data is crucial for clarifying the real oral exposure to contaminants and for establishing appropriate safe levels of benzene in foods.

## Figures and Tables

**Table 1:** Determination of benzene in foods.

Food	Benzene concentration	Country	Reference
Organ meats Fruit-based products, chicken, fish, peanuts, potatoes, vegetable oils	18 ppb <1 ppb	United Kingdom	[43]

Fruits, cheese, eggs, others Roasted peanuts Pickled olives	<1 ppb 1,85 ppb 2,19 ppb	USA	[6]

Carbonated soda beverages (118 samples)	1.1–3.67 ppb	Italy	[44]

Human milk (23 samples)	0.01–0.018 ppm	Italy	[45]

Nonalcoholic and alcoholic beverages	47 samples <1 ppb 20 samples >1.1 ppb–17.6 ppb	Canada	[46]

Nonalcoholic and alcoholic beverages	0.02–18 ppb in the 93 positive products 3 products >5 ppb	Canada	[47]

Carbonated soda beverages (124 samples)	4 samples >5 ppb 2 samples >10 ppb 1 sample = 23 ppb	Canada	[48]

Carbonated soda beverages (134 samples)	Not detected in 33% of the samples Traces in 47% of the samples (0.3 ppb) 10 samples <1 ppb 1 sample >10 ppb	Belgium	[2]

Carbonated soda beverages	77% samples <1 ppb 9% samples >5 ppb	USA	[49]

Carbonated soda beverages (63 samples)	97% of the samples <10 ppb 2 samples >10 ppb	Ireland	[50]

Carrot juice	0.17–2.01 ppb	Germany	[51]

Alcoholic beverages (3 samples)	2.4 ppb in one sample	China	[52]

Carbonated soda beverages	0.15–2.36 ppb	Italy	[53]

Carbonated soda beverages	<1.5 ppb (25°C)	Egypt	[32]

Nonalcoholic beverages (48 samples)	27.08% samples 5.47–16.91 ppb (where 8.33% samples >10 ppb)	Thailand	[54]
